# Impact of Direct Observation on Hand Hygiene Compliance in a Dental University Hospital: A Retrospective Cohort Study

**DOI:** 10.7759/cureus.68827

**Published:** 2024-09-06

**Authors:** Hidetaka Kuroda, Natsuko Y Sawai, Yuki Yamazaki, Hiromi Matsumoto, Hiromi Tsujigami, Shota Tsukimoto, Toshiyuki Handa, Satoshi Ino, Takahiro Abe, Takuro Sanuki

**Affiliations:** 1 Department of Dental Anesthesiology, Kanagawa Dental University, Kanagawa, JPN; 2 Department of Education Planning, Kanagawa Dental University, Kanagawa, JPN; 3 Department of Pharmaceutics, Kanagawa Dental University, Kanagawa, JPN; 4 Department of Nursing, Kanagawa Dental University, Kanagawa, JPN; 5 Department of Oral Hygiene Maintenance, Kanagawa Dental University, Kanagawa, JPN; 6 Department of Dental Anesthesiology, Tokyo Dental College, Tokyo, JPN; 7 Department of Removable Prosthodontics, Kanagawa Dental University, Kanagawa, JPN; 8 Department of Oral and Maxillofacial Surgery, Kanagawa Dental University, Kanagawa, JPN

**Keywords:** alcohol-based hand sanitizers, dental educational hospital, hand hygiene, hand hygiene compliance rate, your 5 moments for hand hygiene

## Abstract

Introduction

Hand hygiene is crucial for preventing healthcare-associated infections in dental settings. Despite its importance, the hand hygiene compliance rates remain unclear, particularly in dental university hospitals where teaching, research, and clinical practice intersect. This study aimed to establish a baseline of hand hygiene compliance rates in a dental university hospital, evaluate the effectiveness of direct observation in improving compliance, and compare practices among different categories of healthcare workers.

Materials and methods

This retrospective cohort study was conducted at Kanagawa Dental University Hospital from April 2022 to March 2023. The design included four blinded direct observations to establish baseline compliance rates, followed by educational training and four unannounced explicit observations. Compliance was assessed based on the World Health Organization's “Your 5 Moments for Hand Hygiene: Dental Care,” adapted for dental outpatient services. The study focused on hand hygiene using alcohol-based hand sanitizers, and compliance rates were calculated for dentists, dental hygienists, dental assistants, and trainee dentists. Monthly consumption of hand sanitizer per patient was tracked from January 2019 as a secondary measure. Statistical analysis included Fisher's exact test, unpaired t-tests, and analysis of variance (ANOVA).

Results

The baseline hand hygiene compliance rate was low at 15.6%, with the highest compliance (25.0%) for “After touching patient surroundings.” Post-intervention, the overall compliance rate increased significantly to 36.0% (p < 0.001). Significant improvements were observed in moments “After body fluid exposure risk” (11.1% to 31.3%, p = 0.004), “After touching a patient” (12.0% to 52.9%, p = 0.006), and “After touching patient surroundings” (25.0% to 73.3%, p = 0.001). Dental hygienists, assistants, and trainee dentists showed significant increases in hand hygiene compliance, while dentists did not. Hand sanitizer consumption increased significantly from 2019 (2.38 ± 0.29 mL per patient) to 2020 (3.47 ± 0.49 mL, p < 0.001) and remained elevated through 2023.

Conclusions

This study revealed low baseline hand hygiene compliance in a dental university hospital setting. While direct observation and education led to significant short-term improvements, especially among allied health professionals, the disconnect between observed compliance rates and hand sanitizer consumption suggests these changes may not represent sustainable behavioral shifts. The varying improvement rates among different healthcare workers and the challenges in maintaining long-term adherence highlight the need for tailored, continuous interventions in dental education and practice settings to enhance hand hygiene compliance.

## Introduction

Hand hygiene is a cornerstone of infection prevention in healthcare settings, and dental practices are no exception. As highlighted by the Centers for Disease Control and Prevention (CDC) in their "Summary of Infection Prevention Practices in Dental Settings: Basic Expectations for Safe Care," proper hand hygiene is crucial in preventing the transmission of harmful pathogens and reducing the risk of healthcare-associated infections (HAIs) in dental settings [1]. While transmission of infectious agents among patients and dental healthcare personnel is relatively rare, documented cases between 2003 and 2015 underscore the importance of stringent infection control practices [1]. These incidents, some involving patient-to-patient transmission, often resulted from lapses in basic infection prevention procedures. Although not always directly linked to hand hygiene failures, these cases emphasize the need for comprehensive infection control measures, with hand hygiene being a fundamental component [1]. The World Health Organization (WHO) stresses that hands are the main pathways for germ transmission during healthcare, and thousands of people die daily from infections acquired while receiving healthcare [2]. In dentistry, where close patient contact and exposure to bodily fluids are routine, the importance of proper hand hygiene cannot be overstated [3].

Despite the understanding that hand hygiene is one of the most effective ways to prevent healthcare-associated infections, actual compliance rates in dental healthcare settings remain insufficient [4]. Given the importance of hand hygiene in dental practice and the potential gap between recommended practices and actual compliance, systematic evaluation and improvement of hand hygiene practices are clearly needed. Interventions to improve hand hygiene compliance include education, the introduction of alcohol-based hand sanitizers, awareness campaigns, voice reminders, hand bacterial cultures, and monitoring and feedback of compliance rates [5]. Among these interventions, direct observation is reported to be superior because it allows assessment of both the timing and quality of hand hygiene implementation [6]. However, there is limited research on hand hygiene compliance and effective interventions in dental school hospitals, which are unique environments integrating teaching, research, and clinical practice. Understanding how factors such as student education and diverse specialty departments impact hand hygiene compliance is crucial for developing effective infection control strategies in these settings.

Therefore, the primary objectives of this study were to (1) establish a baseline of hand hygiene compliance rates in the dental university hospital, (2) evaluate the effectiveness of direct observation as a method of improving hand hygiene compliance in the unique environment of a dental college hospital, and (3) compare hand hygiene practices among different categories of healthcare workers in this setting.

## Materials and methods

Study design and setting

This retrospective study analyzed reports from the infection control team (ICT) at Kanagawa Dental University Hospital, collected between April 2022 and March 2023 in the dental outpatient department. All data, including direct observations of hand hygiene practices and monitoring of alcohol-based hand sanitizer usage, were extracted retrospectively from the routine activity reports of the hospital ICT. All information was anonymized, making it impossible to link the data back to individuals.

The ICT conducted four blinded direct observations to establish a baseline. Following this baseline period, healthcare workers received educational training on hand hygiene via a webinar between October and December 2022. They were informed that further observations would occur, though specific dates were not announced. Subsequently, four unannounced explicit direct observations were conducted.

The dental university hospital's outpatient department includes the following specialties: General Dentistry, Oral and Maxillofacial Surgery, Prosthodontics and Oral Implantology, Periodontics and Endodontics, Pediatric Dentistry, Orthodontics, Special Needs Dentistry, Radiology, and Dental Anesthesiology. To focus on naive dental practice, the departments of Radiology and Dental Anesthesiology were excluded.

The primary outcome measure of this retrospective analysis was the change in hand hygiene compliance rates, calculated for each category of healthcare workers (Dentist, Dental Hygienist, Dental Assistant, and Trainee Dentist). To assess long-term adherence to hand hygiene practices, the study also retrospectively analyzed the consumption of alcohol-based hand sanitizer per patient over the study period as a secondary outcome measure.

Direct observation of hand hygiene compliance

This study focused on hand hygiene practices using alcohol-based hand sanitizers. A single observer monitored the behavior of healthcare workers at 4-6 dental chairs and their surrounding areas. Each observation session lasted 10 ± 5 minutes. The observation criteria were based on the items described in the “Your 5 Moments for Hand Hygiene: Dental Care” (WHO) [7], and each item was redefined as shown in Table 1 to fit the specific workflow of dental outpatient services.

**Table 1:** Observation items

The moments recommended by the WHO	Actual observation items
1. Before touching a patient	Before preparing the dental instruments; Before putting on the cleaning gloves
2. Before clean/aseptic procedure	Before putting on the surgical gloves; Before deploying the dental instruments
3. After body fluid exposure risk	After removing the surgical glove
4. After touching a patient	In situations where it is possible to touch with bare hands
5. After touching the patient's surroundings	After putting away the dental instruments; After removing the cleaning gloves

Hand hygiene compliance rates were calculated by tallying successful and unsuccessful hand hygiene actions for each healthcare worker category and each observation item. The hand hygiene compliance rate was calculated using the following equation: Number of successful hand hygiene actions/Total number of hand hygiene opportunities × 100 (%).

Monitoring of the consumption of alcohol-based hand sanitizer

Since January 2019, Kanagawa Dental University Hospital has been monitoring the monthly consumption of alcohol-based hand sanitizer per patient using the following calculation: Monthly consumption of alcohol-based hand sanitizer in mL/Number of visiting patients per month. We compiled this data annually and compared the consumption across years.

Statistical analysis

Data are expressed as the number of observations (n) or as the mean ± 95% confidence interval (CI). Hand hygiene compliance rates, based on blinded and explicit direct observations, were evaluated using Fisher's exact test. A two-sample independent t-test was used to compare hand hygiene compliance rates among different healthcare worker categories. Trends in the consumption of alcohol-based hand sanitizer were analyzed using one-way ANOVA with Tukey's post hoc test for multiple comparisons. Statistical significance was set at p < 0.05. All statistical analyses were performed using GraphPad Prism 7.05 (GraphPad Software, La Jolla, California).

Ethical considerations

The data on direct observation and monitoring of alcohol-based hand sanitizer consumption in this study were retrospectively extracted from the routine activity reports of the hospital ICT. This information was anonymized in a way that made it impossible to link back to individuals. Therefore, the Kanagawa Dental University Research Ethics Committee determined that this study was exempt from review (accepted number 24-30). This study was registered with the UMIN (University Hospital Medical Information Network) Clinical Trials Registry (UMIN000055152, dated August 3, 2024, available at: https://center6.umin.ac.jp/cgi-open-bin/ctr_e/ctr_view.cgi?recptno=R000063007).

## Results

Baseline and changes in hand hygiene compliance rates

The blinded direct observation reviewed a total of 227 healthcare workers (113 dentists, 47 dental hygienists, 12 dental assistants, and 55 trainee dentists), while the explicit direct observation included a total of 208 healthcare workers (114 dentists, 51 dental hygienists, 17 dental assistants, and 26 trainee dentists). Note that not all hand hygiene moments were observed for every healthcare worker in either observation period.

To establish a baseline of hand hygiene compliance rates in the dental university hospital and evaluate the effectiveness of direct observation in improving hand hygiene compliance, we assessed compliance rates during both blinded and explicit direct observations. The results are shown in Table 2. Across all healthcare worker categories and all hand hygiene moments, the hand hygiene compliance rate during blinded direct observations was 15.6%. Among the hand hygiene moments, compliance was highest for the "After touching patient surroundings" category, at 25.0%. After conducting educational training, the compliance rate significantly increased to 36.0% during explicit direct observations (odds ratio (OR): 0.33, 95% CI: 0.21-0.50, p < 0.001). The increase was particularly significant for "After body fluid exposure risk" (from 11.1% to 31.3%, OR: 0.27, 95% CI: 0.12-0.67, p = 0.004), "After touching a patient" (from 12.0% to 52.9%, OR: 0.12, 95% CI: 0.03-0.59, p = 0.006), and "After touching patient surroundings" (from 25.0% to 73.3%, OR: 0.12, 95% CI: 0.04-0.38, p = 0.001).

**Table 2:** Results of direct observations for all healthcare workers Data are expressed as the number of observations (n). Hand hygiene compliance rates (HCR) (%) were calculated using the equation described in the Methods section. Note that OR < 1 indicates higher odds of success in explicit observation compared to blinded observation. The inverse of the OR (1/OR) represents the increased odds of success in explicit observation. Statistically significant differences are indicated by asterisks. * *p* < 0.05. n.d. denotes no data available, OR: odds ratio; 95% CI: 95% confidence interval.

	Blinded direct observations		Explicit direct observations		OR (95% CI)	P values
	Success (HCR (%))	Failure	Success (HCR (%))	Failure		
Overall	40 (15.6)	216	83 (36.0)	147	0.33 (0.21-0.50)	<0.001*
1. Before touching a patient	9 (19.1)	38	12 (29.3)	29	0.57 (0.22-1.58)	0.320
Dentist	6 (33.3)	12	4 (17.3)	19	-	-
Dental hygienist	0 (0)	12	5 (50)	5	-	-
Dental assistant	0 (0)	2	1 (33.3)	2	-	-
Trainee dentist	3 (20)	12	2 (40)	3	-	-
2. Before clean/aseptic procedure	10 (14.9)	57	19 (25.3)	56	0.52 (0.22-1.17)	0.147
Dentist	3 (8.5)	32	5 (12.5)	35	-	-
Dental hygienist	2 (15.3)	11	7 (50)	14	-	-
Dental assistant	0 (0)	3	4 (66.7)	2	-	-
Trainee dentist	5 (31.2)	11	3 (37.5)	5	-	-
3. After body fluid exposure risk	9 (11.1)	72	21 (31.3)	46	0.27 (0.12-0.67)	0.004*
Dentist	5 (10.2)	44	13 (33.3)	26	-	-
Dental hygienist	1 (7.1)	13	4 (23.5)	13	-	-
Dental assistant	0 (0)	3	2 (40)	3	-	-
Trainee dentist	3 (20.0)	12	2 (33.3)	4	-	-
4. After touching a patient	3 (12.0)	22	9 (52.9)	8	0.12 (0.03-0.59)	0.006*
Dentist	1 (7.1)	13	4 (50)	4	-	-
Dental hygienist	0 (0)	5	1 (33.3)	2	-	-
Dental assistant	n.d.	n.d.	1 (100)	0	-	-
Trainee dentist	2 (33.3)	4	3 (60)	2	-	-
5. After touching the patient's surroundings	9 (25.0)	27	22 (73.3)	8	0.12 (0.04-0.38)	0.001*
Dentist	5 (29.4)	12	9 (64.3)	5	-	-
Dental hygienist	1 (20)	4	8 (80)	2	-	-
Dental assistant	2 (40)	3	4 (100)	0	-	-
Trainee dentist	1 (11.1)	8	1 (50)	1	-	-

**Figure 1:**
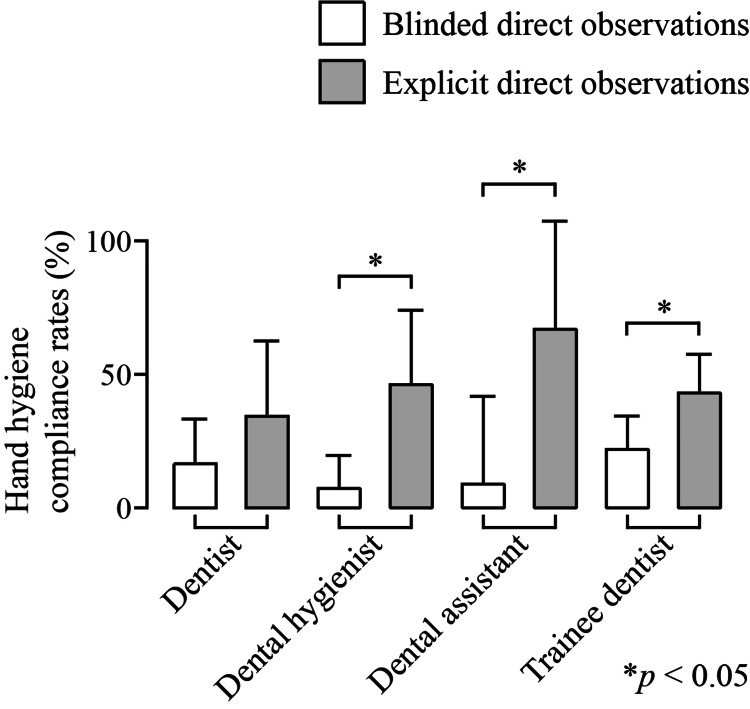
Comparison of hand hygiene compliance rates among different healthcare worker categories The overall hand hygiene compliance rates (%) for each healthcare worker category are presented. Compared to blinded direct observation (white boxes), explicit direct observation (gray boxes) resulted in a significant increase in hand hygiene compliance rates among healthcare workers, with the exception of dentists. Data are expressed as the mean ± 95% confidence interval (CI). Statistically significant differences are indicated by asterisks (* p < 0.05).

In the evaluation by categories of healthcare workers, explicit direct observation showed a significant increase in hand hygiene compliance rates compared to blinded direct observation. The compliance rate for dental hygienists increased from 8.48 ± 11.19% to 47.36 ± 26.68% (p = 0.006), for dental assistants from 10.0 ± 31.82% to 68.0 ± 39.4% (p = 0.02), and for trainee dentists from 23.12 ± 11.33% to 44.16 ± 13.38% (p = 0.01) (Figure 1).

Trends in the consumption of alcohol-based hand sanitizer

**Figure 2:**
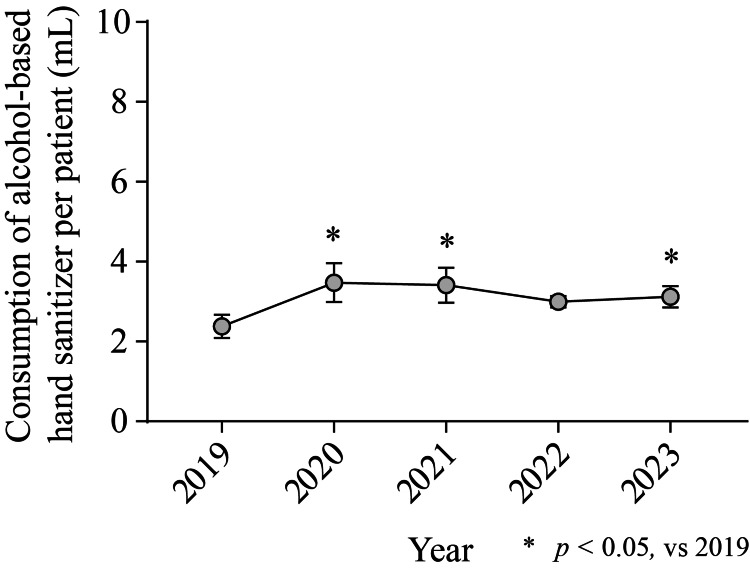
Trends in the consumption of alcohol-based hand sanitizer The figure represents the annual consumption of alcohol-based hand sanitizer per patient. The consumption was calculated as the mean of monthly usage amounts (gray circles), determined using the equation described in the Methods section. Data are expressed as the mean ± 95% confidence interval (CI). Statistically significant differences are indicated by asterisks (* p < 0.05).

To evaluate long-term adherence to hand hygiene practices, we assessed the annual consumption of alcohol-based hand sanitizer per patient. In 2019, the consumption per patient was 2.38 ± 0.29 mL (Figure 2). Compared to 2019, the consumption per patient significantly increased in 2020 (3.47 ± 0.49 mL, *p* < 0.001), 2021 (3.41 ± 0.46 mL, *p* = 0.001), and 2023 (3.12 ± 0.27 mL, *p* = 0.014).

## Discussion

This study revealed that the baseline hand hygiene compliance rate at the dental university hospital was approximately 15%. This rate was improved through direct observation, which proved particularly effective for allied health professionals such as dental hygienists, dental assistants, and trainee dentists. However, sporadic direct observations might not have a significant impact on long-term improvement in hand hygiene compliance rates.

A study conducted in a Moscow municipal hospital, which complied with WHO guidelines, reported that implementing an action plan significantly increased hand hygiene compliance among healthcare personnel from 52.3% to 83.3% over a three-month period, with nurses showing the highest compliance rate at 79.6% [8]. Similarly, hand hygiene compliance in a Finnish tertiary-care hospital increased significantly from 76.4% to 88.5% following the introduction of direct observation and feedback [9]. Although these reports showed improvements from a high baseline, they were conducted in medical hospitals where the nature of care may differ from dentistry. A national survey of Canadian dentists in 1995 found that while compliance with some infection control practices was high (e.g., 95% always wearing gloves), there was significant room for improvement in handwashing, with only 76% washing hands before treating patients and 63% after removing gloves [10]. A study of postgraduate year dentists at a Taipei university teaching hospital reported an overall handwashing compliance rate of 34.7%, with significant differences between oral surgery services (92.8%) and general clinical practice (34.2%) [11]. The accuracy of handwashing was also higher in oral surgery (87.5%) compared to general practice (51.0%) [11], indicating a need for improved education and monitoring of hand hygiene practices among dentists, particularly in general clinical settings.

In this study, the baseline hand hygiene compliance rate was 15.6% overall (Table 2), which is relatively low compared to other reports. While a previous study reported higher hand hygiene compliance rates in areas with a high contamination risk and lower rates in low-risk areas such as patients’ personal items [12], our study showed contradictory results, with low compliance rates even after body fluid exposure risk (31.3% under explicit direct observations) (Table 2). Notably, this study focused on hand hygiene practices using alcohol-based hand sanitizers. Studies have shown that most dental practitioners frequently use soap and water for hand hygiene, while fewer regularly use alcohol-based hand sanitizers [13,14]. If instances of handwashing with soap and water had been included in the hand hygiene count, the compliance rates in this study (including the baseline) might have been higher.

The low baseline hand hygiene compliance rates observed in this study raise questions about the underlying causes of poor adherence. Previous studies have identified a lack of knowledge regarding proper hand hygiene among dentists [4], suggesting that insufficient training may be a contributing factor. However, other research indicates that even when dental healthcare workers possess this knowledge, outbreaks of severe infections have not significantly improved their hand hygiene practices [15]. This paradox suggests that the issue may not solely be a lack of knowledge, but rather a combination of factors including insufficient emphasis on the importance of hand hygiene in dental education, inadequate reinforcement of learned practices, and potential barriers to implementation in clinical settings. Future research should aim to disentangle these factors to develop more effective educational and practical interventions for improving hand hygiene compliance in dental university hospitals.

This study revealed a paradox: while observed hand hygiene compliance more than doubled (from 15.6% to 36.0%) (Table 2), the consumption of alcohol-based sanitizer remained static (Figure 2). This disconnect raises questions about the long-term efficacy of interventions and the reliability of observational data in reflecting actual daily practices. It suggests that the short-term improvement may be more attributable to the Hawthorne effect (the phenomenon where behavior changes when people are aware they are being observed) than to genuine behavioral change, underscoring the challenges in sustainably improving hand hygiene adherence in healthcare settings. The Hawthorne effect is a psychological phenomenon where individuals modify their behavior in response to being observed [16]. Generally, this effect can bias study results, particularly in behavioral and social studies. However, if we consider the Hawthorne effect as a strategic tool to induce behavioral change in healthcare workers, repeated explicit direct observations and interventions may help transform temporary behavioral changes into long-term habits.

When comparing compliance rates across different healthcare professions, dental hygienists, dental assistants, and trainee dentists showed significant increases in hand hygiene adherence (Figure 1). Observational longitudinal studies conducted in dental educational institutions consistently report higher hand hygiene compliance among dental educators and established dentists compared to students, residents, and other dental professionals [17-20]. Notably, dental educators demonstrated the highest compliance rates (63.7-78.4%), while students and residents consistently fell below 50%. This disparity suggests that experience and knowledge of healthcare-associated infections (HAIs) may significantly influence adherence to hand hygiene protocols in dental settings. While the Hawthorne effect cannot be entirely ruled out, the increased hand hygiene compliance observed among non-dentist professionals in this study may also be attributed to the effectiveness of the educational training on hand hygiene that was conducted.

This study has several limitations. First, the consumption of alcohol-based hand sanitizers significantly increased after 2020 (Figure 2). This increase may be attributed to the COVID-19 pandemic, which began in January 2020. Indeed, the outbreak improved hand hygiene practices in general dental clinics [21]. Therefore, it is highly likely that this increase in sanitizer usage was driven more by the pandemic than by direct observation or educational training. 

Second, comparing our results with previous studies poses a challenge. Various biases, such as observation bias, selection bias, information bias, and the Hawthorne effect, can influence hand hygiene monitoring outcomes. When publishing compliance data, it is recommended to report not only the total number of hand hygiene opportunities but also the breakdown of opportunities and actions for each of the five moments for hand hygiene [22]. In line with this recommendation, our study collected and reported data for each of the five moments of hand hygiene. However, many of the studies cited in our discussion primarily focused on the total number of hand hygiene opportunities, making direct comparisons difficult. If previous studies had broken down their data into the five moments, their reported compliance rates might have decreased significantly. Still, our results cannot be described as having high compliance rates. 

Additionally, the low consumption of alcohol-based sanitizer observed in this study suggests that insufficient amounts might have been used per hand hygiene event. Interventions to improve hand hygiene compliance rates include educational training, compliance monitoring with feedback, visual reminders, and auditory cues to prompt action. Combining these interventions with continuous awareness-raising and recognition has been reported to be effective in improving compliance rates [5]. In the unique environment of a dental university hospital with affiliated educational institutions, further consideration is needed to determine the most appropriate methods for improving hand hygiene compliance rates.

## Conclusions

This study revealed low baseline hand hygiene compliance rates in a dental university hospital setting, with significant room for improvement across all healthcare worker categories. While direct observation led to short-term improvements, particularly among allied health professionals, the lack of a corresponding increase in alcohol-based sanitizer consumption suggests these changes may be more attributable to the Hawthorne effect than to sustainable behavioral shifts. However, it is important to note that the Hawthorne effect, when strategically leveraged, can contribute positively to behavioral change. Further research and tailored interventions are necessary to address the unique challenges in dental university hospitals and to develop effective strategies for long-term improvement in hand hygiene compliance.
